# The Drainage of Interstitial Fluid in the Deep Brain is Controlled by the Integrity of Myelination

**DOI:** 10.14336/AD.2018.1206

**Published:** 2019-10-01

**Authors:** Aibo Wang, Rui Wang, Dehua Cui, Xinrui Huang, Lan Yuan, Huipo Liu, Yu Fu, Lei Liang, Wei Wang, Qingyuan He, Chunyan Shi, Xiangping Guan, Ze Teng, Guomei Zhao, Yuanyuan Li, Yajuan Gao, Hongbin Han

**Affiliations:** ^1^Department of Radiology, Peking University Third Hospital, Beijing, China.; ^2^Key Laboratory of Magnetic Resonance Imaging Equipment and Technique, Beijing, China.; ^3^Department of Biophysics, School of Basic Medical Sciences, Peking University, Beijing, China.; ^4^Peking University Medical and Health Analysis Center, Peking University Health Science Center, Beijing, China.; ^5^Institute of Applied Physics and Computational Mathematics, Beijing, China.; ^6^Department of Medical Chemistry, School of Pharmaceutical Sciences, Peking University, Beijing, China.; ^7^Department of Neurology, Peking University Third Hospital, Beijing, China.

**Keywords:** interstitial fluid, extracellular space, tracer-based magnetic resonance imaging, myelination

## Abstract

In searching for the drainage route of the interstitial fluid (ISF) in the deep brain, we discovered a regionalized ISF drainage system as well as a new function of myelin in regulating the drainage. The traced ISF from the caudate nucleus drained to the ipsilateral cortex along myelin fiber tracts, while in the opposite direction, its movement to the adjacent thalamus was completely impeded by a barrier structure, which was identified as the converged, compact myelin fascicle. The regulating and the barrier effects of myelin were unchanged in AQP4-knockout rats but were impaired as the integrity of boundary structure of drainage system was destroyed in a demyelinated rat model. We thus proposed that the brain homeostasis was maintained within each ISF drainage division locally, rather than across the brain as a whole. A new brain division system and a new pathogenic mechanism of demyelination are therefore proposed.

Understanding substance transportation in the extracellular space (ECS) of the brain is essential for understanding how the brain functions in the absence of a lymphatic drainage pathway [1-5]. The ECS is the immediate environment surrounding neurons and glial cells and contains interstitial fluid (ISF) and the extracellular matrix (ECM) network [2, 6]. The ISF is secreted by the cerebral vascular endothelium, or blood-brain barrier and water, ions, gaseous molecules and organic molecules such as proteins, peptides, enzymes, dopamine, extracellular vesicles consist its main ingredients [7]. It fills the ECS and is the medium for nutrients supply, waste removal and intercellular communication, which is pivotal in maintaining the brain homeostasis [8, 9]. It delivers metabolites, neurotransmitters, and drug agents to target brain regions to exert their functions [2]. Immunofluorescence studies have revealed the ISF drainage route in the superficial cortex and its migration to the deep cervical lymph nodes via lymphatic vessels lining the dural sinuses, leading to the view that the brain ECS is a highly connected system [1, 2, 10]. Brain ISF drainage in deep nuclei was recently studied using a newly developed method of tracer-based MRI, whereby ISF drainage in the deep brain appeared to be regionalized with various distribution territories and diffusion rates in different locations [11]. Notably, the traced ISF from the thalamus (Tha) could not drain to the ECS of the caudate nucleus (Cn) and vice versa, even though these two regions are adjacent to one another [11-13]. A transportation barrier to brain ISF drainage thus appears to exist in the deep brain. Confirmation of this separation effect and identification of the barrier structure would imply that brain homeostasis is maintained locally within each ECS division rather than at a whole-brain scale. As such, verifying the phenomenon will help to elucidate the glymphatic drainage pathway in the deep brain and will be crucial for future applications in neuroscience [12, 14, 15].

In this study, regionalized ISF drainage in the deep nuclei was reconfirmed with laser scanning confocal microscopy (LSCM) using a fluorescent probe (Lucifer Yellow, LY) in male Sprague-Dawley rats. The stability of the separation effect on ISF drainage was tested and analyzed on aquaporin-4 (AQP-4) knockout rat group. After the transport separation was verified using both optical and magnetic resonance imaging methods, the structure impeding the drainage was identified as myelinated fibre tracts using electron microscopy and immunohistochemical staining (Black Gold and Fast Blue) under light microscopy. We examined changes in ISF drainage properties in a demyelinated model to further validate the effects of myelin in regulating brain ISF drainage and its significance in maintaining local brain homeostasis.

## MATERIALS AND METHODS

### Animals

This study was performed in accordance with the national guidelines for the use of experimental animals, and the protocols were approved by the Ethics Committee of Peking University Health Center.

### AQP4 knockout rats

Adult male Sprague-Dawley rats weighing 250-300g were used. The transcription activator-like effector nuclease (TALENs)-mediated knockout approach was performed to generate aquaporin 4 (AQP4)-deficient rats, as previously described. Briefly, we designed and synthesized highly active TALENs against the following sequences: (5’-CACAGCAGAGTTCCTGG-3’) for the sense strand and (5’-GGATCCCACGCTGAGCA-3’) for the antisense strand. The mRNAs of TALENs were injected into the cytoplasm of rat pronuclear stage embryos to produce mutant founders (F0). F0 rats, which lacked three base pairs, were crossed with wild-type rats to produce the F1 generation. The heterozygous offspring of F1 rats were crossed to generate F2. Genomic analysis, conducted by sequencing of PCR products, showed that the pups were heterozygous. The AQP4^-/-^ rats were viable, fertile, and did not exhibit any gross abnormalities. This finding indicates that the TALEN-introduced AQP4 mutation was stably inherited.

### Demyelinated rats

Adult male Sprague-Dawley rats weighing 250-300g were used. Eight-week-old male SD rats were maintained on a diet enriched with 1% cuprizone and 0.025% blue food colour by dry weight (Envigo, Indianapolis, IN, USA), *ad libitum*, for 4 weeks. The effects of cuprizone-induced demyelination were confirmed by EM and Black-Gold staining (Fig. 3).

### Imaging analysis method

Tracer-based MRI (NanoDetect Analyze system (MRI lab; Beijing, China; version 2.1)

The detail information can be found at see supplementary Fig. 1-3.

Stereotaxic intracranial injections of tracer in tracer-based MRI

The tracer, Gd-DTPA (Magnevist; Bayer Schering Pharma AG, Berlin, Germany) or an optic-magnetic bimodal molecular tracer, Gd-DO3A-ethylthiouret-fluorescein (Gd-DO3A-EA-FITC), which was synthesized by our lab, was diluted to 10 mmol/L with 154 mmol/L NaCl solution. After the injection of 2 μL (10 mmol/L) to different regions of the brain, tracer concentration decreased rapidly in a short period of time. These tracers also cannot be uptaken by neuroglial cells. MRI scanning was used to conduct and design the injection route and depth before injection. Each rat was anesthetized with intraperitoneal injection of sodium pentobarbital (50 mg/kg); core temperature was monitored with a rectal thermometer and maintained with a heating pad at 38 ± 0.5°C. Additionally, other physiological variables, such as blood pressure, heart rate and respiratory rate, were monitored; these showed no significant differences between the groups (data not shown). The skin covering the calvaria was shaved and disinfected with iodized alcohol. An incision was made in the scalp along the sagittal suture from the interaural area to the interocular area. The membranes and muscle attachments were dissected free of the skull bone, and the bregma suture was exposed. The rat was immobilized in a stereotactic coordinate system (Lab Standard Stereotaxic-Single, Stoelting Co., Wood Dale, IL, USA) and a small trephine hole was made in accordance with the stereotactic coordinates of Tha (bregma: -3.0 mm, lateral: 2.0 mm, vertical: 6.0 mm) or *Cn* (bregma: +1.0 mm, lateral: 3.5 mm, vertical: 5.0 mm). A 2-μl total volume of the tracer (10 mmol/L) was delivered into the brain interstitial space (ISS) via a 10 μL microsyringe (Hamilton, Bonaduz AG, Switzerland) at a rate of 0.2 μL/min with an automated drug administration system (Harvard Apparatus, Holliston, MA, USA), following a 5-min waiting. The rat was then quickly placed in the scanner in a prone position for the post-injection scan, in accordance with the MRI scan protocols.

#### MRI scan protocol

A 3.0 T MRI system (Magnetom Trio, Siemens Medical Solutions, Erlangen, Germany) with an eight-channel wrist coil was used to obtain brain images by T1-weighted magnetization-prepared rapid-acquisition with gradient echo (MP-RAGE) sequence. The acquisition parameters were as follows: echo time = 3.7 ms, repetition time = 1500 ms, flip angle = 12°, inversion time = 900 ms, field of view = 267 mm, voxel = 0.5 mm^3^, matrix = 512 × 512, number of averages = 2, phase-encoding steps = 96. The acquisition time for each rat was 290 s. For each subject, scanning was performed before and after the introduction of tracer. The scan time points were set as pre-injection and each post-injection time point until the “bright region” faded.

#### Post-procedure calculations of physiological parameters

MATLAB-based software was developed to co-register the MR images before and after the injection. All images following the injection were automatically subjected to rigid transformation, similarity measurements, high-order interpolation and an adaptive stochastic gradient descent optimization. These images were then subtracted from the pre-scanned image. The acquired “bright areas”, which were obtained by establishing a seed point, and a threshold in the ROI were assumed to be related to the tracer. New sets of post-processing MR images with slice thicknesses of 1 mm were generated by the software. After the co-registration and subtraction processes were completed, signal intensity within the target area of the processed images was measured and denoted by ∆SI, which was used to calculate the diffusion parameter in the ECS of brain.

Brain tissue around the injection site appeared as high-intensity spot on the image, after the injection of tracer. The enhancement of the MR signal intensity caused by the tracer was converted to its concentration by an empirical fitting process. Thus, both the flow and diffusion parameters of the brain interstitial fluid (ISF) could be calculated on the basis of the obtained distribution of the tracer concentration. By the modified diffusion equation, the equivalent diffusion coefficient, D, and clearance parameters, k’, in each MRI pixel near the injection site could be derived. Here, Vdmax is defined as the ratio of the maximum volume distribution of traced ISF to the total brain volume, measured by the above-described method. Because the clearance of tracer in the whole brain fit well with a mono-exponential decay function, the k’ and half-life (t½) could be used to represent both clearance rate and transportation speed.

After obtaining MR images of the brain, we drew a three-dimensional (3D) wireframe by the “matplotlib” Python package. The height of the figure represents the signal intensity of the MR image. The XY plane at the bottom of the figure is a contour map, superimposed on the original MR image.

We also produced a 3D transparent model of the brain via volume-rendering technology. First, the complete brain was artificially segmented from MR images. Then, based on the segmented brain MR image, the 3D transparent model was generated using the VTK toolkit. We used red colour to represent the injection area in the model.

#### Mathematical modelling

Based on the modified diffusion (1) and a standard least-squares fitting technique (2), a program (unpublished) was used for the computation of the diffusion parameters as follows:
(1)
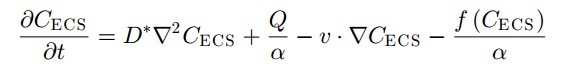
where C_ECS_ is the actual concentration in the extracellular space (ECS) and is a function of time (t) and position. The signs ? and ?^2^ symbolize, respectively, the first and second spatial derivatives in the appropriate coordinate system. The volume fraction α is defined as the volume fraction of ECS in the whole brain tissue. Tortuosity is defined as λ = (D/D*)^1/2^, where D* is the effective diffusion coefficient of a given molecule in the brain ECS and D is the diffusion coefficient of the same molecule in a free medium. λ is the hindrance to diffusion, imposed by the local structure of the ECS; Q is the source that is released into the ECS. In the following text, the term “source” refers to the initial concentration available for diffusion or in the case of an exogenous tracer (e.g., Gd-DTPA), it refers to the site of the injection. Because there was no constant input to the system, Gd-DTPA was assumed to have been administered at the original time point; thus, the source Q can be ignored in the present study. v represents the flow rate of the interstitial fluid in the ECS in mm/s. We set the ROI as close to the injection point as possible (2 mm from the original point), and bulk flow is neglected because of its minimal influence on diffusion. ISF in the local ECS is also assumed to be nondirectional. We further deduced the following equation to facilitate the computation of the diffusion parameters:

(2) $$\text{χ}=\overset{N}{\underset{t=1}{\sum }}\overset{r_{0}}{\underset{0}{\int }}\Delta SI\left(t_{i},r;D^{*},\beta \right)-\Delta SI,m{\left(t_{i}.r\right)}^{2}dr$$

Where ΔSI,_m_ (t_i_,r) represents the measured radial profile at time t_i_: as a series of MR images are captured at predetermined time intervals, t_i_ (i = 1, 2, ..., n), the radial profile along a given radial path for each MR image can be measured. The diffusion parameters D* and β can be measured by matching the model and measured profiles at time points t_i_. In the present study, the “matching” processes were performed by the standard least-squares fitting technique; the function variable χ was minimized to find D* and β, and the error of the measured profile was assumed to be the same for all data points. In the computation of χ, the integration over radius r was replaced by summation along a given path, which was a particular axis along which the measurements were made from the injection site. A simplex-downhill method was used to perform the numerical minimization.

### Laser scanning confocal microscopy

#### Preparation of brain slices

The oblique sagittal brain slices were prepared to facilitate simultaneous viewing of the caudate nucleus, internal capsule, and thalamus in the same slice.

The oblique sagittal slice was rotated 15° outward from the sagittal position, using the midpoint of the cerebellum as its axis. In this section, we could simultaneously observe the caudate nucleus, internal capsule, thalamus, and their associated fibre tracts. It was also convenient for observing ISF drainage by tracer-based imaging methods (Supplementary Fig. 4).

#### The fluorescent tracer and scanning protocol

Lucifer yellow CH (Sigma, St. Louis, MO, USA) was selected as a fluorescent tracer for in vivo measurements from a series of fluorescent tracers, including dextran-tetramethylrhodamine (Invitrogen, Carlsbad, CA, USA), acridine orange (Sigma), and Gd-DO3A-ethylthiourea-FITC. A concentration of 10 mmol/L Lucifer yellow CH was diluted with 154 mmol/L artificial cerebrospinal fluid; the procedure for stereotactic injection was similar to that used for Gd-DTPA. Rats were perfused transcardially with 300 mL physiological saline for 15 min, followed by 150 mL 4% paraformaldehyde. Brains were removed and post-fixed in 4% paraformaldehyde (6 h, 4°C). Brain slices were made along the connection direction of caudate nucleus and thalamus.

The Leica TCS SP8 MP FLIM new Merging function can store m × n single images during the process of scanning. The whole image of the sample in the designated area can then be obtained by image reconstruction after scanning. Experimental fluorescence images were collected by the LSCM Merging function. LSCM parameters: objective, 5× and 25×; LY wavelength: λEx = 488 nm, λEm = 530 ± 20 nm.

### DTI scan protocols using 7.0T MRI

A spin-echo echo-planar diffusion tensor imaging (DTI) sequence was used with the following acquisition parameters: repetition period (TR) of 5,000.001 ms, echo time (TE) of 26 ms, flip angle of 90°, time between application of gradient pulses (Δ) of 14 ms, diffusion gradient duration (δ) of 3 ms, slice thickness of 0.8 mm, slice interval of 0 mm, field of view of 30 × 30 mm^2^, and matrix size of 128 × 128 (zero filled to 256 × 256). Diffusion-sensitizing gradients were applied along 30 directions. The b-value used for the acquisition of diffusion-weighted images was 800 s/mm^2^. The nominal voxel size was 117 × 117 × 800 μm after zero filling. The scanning area was set ranging from the genu of the corpus callosum (CC) to the end of medulla oblongata; the total imaging time was approximately 46 min, 40 s, 0 ms.

Using a region-of-interest (ROI)-based approach, DTI parameters (λ1, λ 2, λ 3, functional anisotropy (FA), and apparent diffusion coefficient (ADC)) were acquired from the CC, IC, EC, and PY through 20 coronal slices for the image sets with the same anatomical landmark-based rules. FA images were used for outlining the ROIs. The image software (Paravision 5.0) binding feature allowed for the replication of the traced ROI to other images.

### Electron microscopy

#### Preparing fresh brain tissue for cryofixation

After rats were deeply anesthetized with intraperitoneal injection of sodium pentobarbital (50 mg/kg), trepanation with a bone drill and biopsy sampling with a biopsy needle were conducted under the guidance of a brain stereotaxic apparatus. For our experimental purposes, the junction tissues among the caudate nucleus, ipsilateral cortex, and thalamus were collected.

#### Cryofixation with high-pressure freezing

After each biopsy, the brain tissue was then further sliced into pieces with a razorblade, in order to fit into the 200-μm deep cavity of the 3-mm diameter aluminium platelets used for high pressure freezing. Then, a small drop of 1-hexadecene was added to fill the space of the cavity not occupied by the specimen, in order to avoid air bubbles trapped inside the specimen. The specimen sandwich was completed by the flat side of another platelet. The sandwich was then high-pressure frozen by a Leica EM HPM 100 (Leica Microsystems). The frozen cryoimmobilized sample was immediately transferred into liquid nitrogen until further processing. This entire procedure was completed as rapidly as possible (in less than 90 s) after removing the biopsy needle.

#### Freeze substitution of high-pressure frozen samples

The frozen sample was removed from the specimen sandwich and all samples were transferred into Eppendorf tubes filled with anhydrous acetone containing 1% osmium tetroxide and 0.1% uranyl acetate, precooled to -90˚C. Then, frozen tissues were stained, dehydrated, and embedded at low temperature by an automatic freeze-substitution unit (AFS2, Leica Microsystems). The samples were kept at -90˚C, -80˚C, -60˚C, and -30˚C in sequence for 8 h at each step and finally brought up to 0˚C for 2 h, whilst the temperature rose to 21˚C.

#### Resin embedding and sample selection

After 3 washes with anhydrous acetone at 15-min intervals, the samples were embedded in the conventional manner and were infiltrated with Embed-812 resin: 100% EtOH (1:2, 1:1, 2:1; 1 h each) and 24 h at 65˚C in fresh 100% resin after two changes in 100% Embed-812. Cryo-fixed tissue that showed no artefacts of fixation, such as ice crystal damage, was selected by cutting semi-thin (0.5-mm thick) sections (2 × 2 mm) of the resin-embedded material. With transmitted light microscopy, an area of well-fixed tissue could be identified; these regions were further trimmed and either used for TEM or FIB-SEM.

#### TEM imaging

Ribbons of sections sliced to 50 nm thick with an ultramicrotome (Leica EM UC7) were mounted onto single-slot copper grids holding Formvar support film. These sections were examined under a Tecnai Spirit (FEI Company) 120 kV transmission electron microscope with an Eagle 4k × 4k CCD camera.

#### FIB-SEM imaging

The serial section images were viewed in conjunction with the TEM image that had been previously examined and served as references to identify the same myelin fascicles. The trimmed resin tissue blocks were mounted with conductive paint to aluminium stubs and gold coated (Cressington), then placed inside Helios G4 UC FIB-SEM (FEI Company). After ion milling to expose the required region, an imaging electron beam of 1.6 kV was used with a milling beam of 30 kV and 800 pA. Each milling and imaging cycle took approximately 90 s, with a pixel dwell time for the electron beam of 10 ms. Six hundred to one thousand images were collected by sequentially milling and imaging, with final image sizes of 2048 × 1536 pixels. Each pixel was 5 × 5 nm.

#### Image 3D reconstruction and analysis

Serial FIB-SEM images were reconstructed by the free open-source software packages, such as IMOD (http://bio3d.colorado.edu/imod/), or our custom-made software. Models of myelin fascicles segmented from reconstructed volume were then imported into the 3D modelling software Blender (www.blender.org) for final composition, rendering, and analysis.

### Histological staining

#### Luxol Fast Blue and H&E staining

Myelin was studied with Luxol Fast Blue (LFB) staining, which binds to the bases of the lipoproteins in the myelin sheath. The staining was performed by following Hans B. Snyder's protocol in the Armed Forces Institute of Pathology manual. Briefly, after fixation, spinal cord specimens were embedded in paraffin, cut into 8-10 μm oblique sagittal sections, dried at 37°C overnight, de-paraffinized, cleared, then hydrated in 95% EtOH, rinsed in distilled water, and stained in 0.1% LFB. Slides were rinsed in 95% EtOH to remove excess stain, then in water, differentiated in 0.05% lithium carbonate and 70% EtOH, and counterstained with haematoxylin and eosin (H&E). Finally, sections were dehydrated, cleaned in xylene, and mounted. Upon LFB staining, myelin fibres appeared blue, neuropils appeared pink, and neural cell bodies appeared purple. Slices were viewed, and images were captured by an Olympus microscope equipped with MagnaFire SP image acquisition software. Images were formatted with Adobe Photoshop software.

#### Black Gold and Nissl’s staining

Sections were washed three times for 10 min in cold KPBS, then mounted onto Superfrost slides and dried overnight at 25? temperature. Each slide contained samples from each experimental condition. All slides were stained concurrently. Slides were rehydrated in water for 2 min, then transferred into a slide mailer tube (Simport) containing 10 mL of 0.3% Black Gold II (AG105, Millipore) aurophosphate stain diluted in 0.9% NaCl preheated to 64°C. Slides were incubated for exactly 16 min at 64°C, then rinsed twice in reverse osmosis-purified (RO) water for 2 min each. Slides were then incubated in a preheated 1% sodium thiosulfate solution for 2 min at 64°C, washed three times in RO water for 2 min each, dehydrated, and coverslipped with xylenes and Permount. For Nissl staining, frozen sections were hydrated by rinsing into 100%, 95%, 90%, 80%, and 70% alcohol, then distilled water, in order (5 min each). Then, the sections were stained in a 1% toluidine blue solution for 5-10 min, differentiated in 75% alcohol for seconds, and rinsed quickly in distilled water.

**Figure 1.**
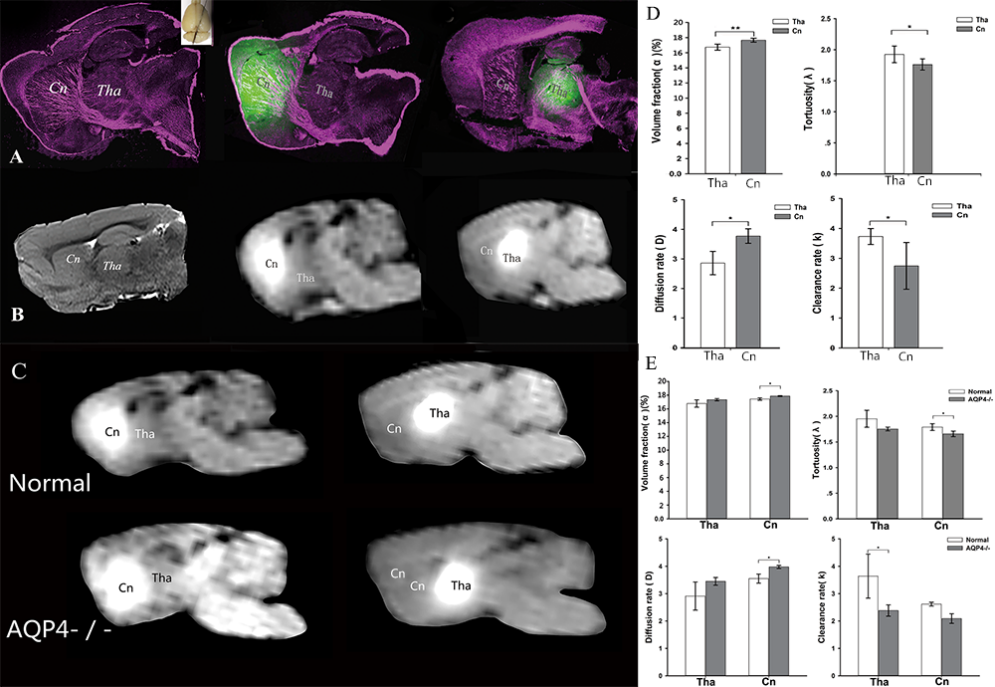
Evidence for a transport barrier between ISS divisions. A) LSCM conducted after fluorescent tracer was injected into Cn and Tha. After injection, tracer distributions were limited within the two divisions. B) The transportation barrier between Cn and Tha with MRI in oblique-sagittal slices. Right panel: a high-resolution T2-weighted image. Middle and left panels: tracer-based dynamic T1-weighted images in which the tracer distribution reached maximum volumes. After paramagnetic tracer injection, the local tissue appeared as a hyper intense spot on MR. In Cn, tracer distribution was more extensive and the traced ISF flowed to the ipsilateral frontal cortex. No distribution was observed in Tha. The enhancement in Tha was localized to its anatomical division and didn’t flow to Cn. In the AQP4-knockout rats the barrier effect was still stable (C). The structural and functional parameters in different ISS divisions were disparate (D). The volume fraction was higher in Cn than Tha (P <0.01). The tortuosity was lower in Cn than Tha (P <0.05). The clearance rate was lower in Cn than Tha (P <0.05). The diffusion rate was higher than that in Tha (P <0.05). The structural and functional parameters of ISS in AQP4-knockout rats were disparate (E). In Tha, the volume fraction, tortuosity, and diffusion rate were not statistically different between AQP4-knockout and control groups. The clearance rate was lower in AQP4-knockout group than control group (P <0.05). In Cn, the volume fraction in AQP4-knockout group was higher than that in control group (P <0.05) and the tortuosity was lower than control group (P <0.05). The clearance rate in AQP4-knockout group was lower than that in control group (P <0.05), while the diffusion rate was higher than control group (P <0.05).

**Figure 2.**
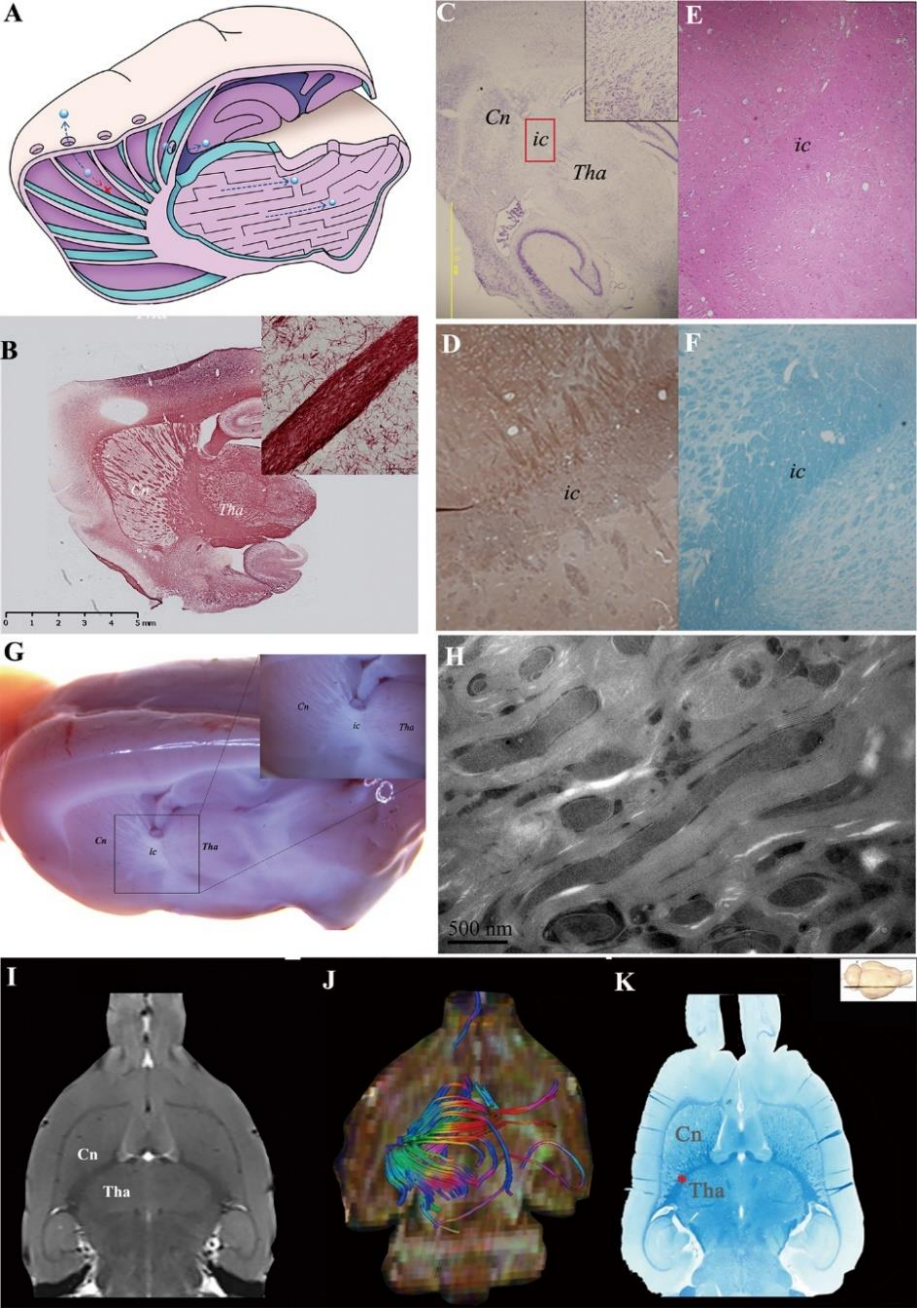
Barrier structure was identified as compact myelin fibre tracts. (A) denotes the transportation barrier between the ISS of the Cn and Tha in control group. The ISF (blue ball) in the Cn flows to the ipsilateral frontal cortex. The ISF in the thalamus is localized in its anatomical division. The communication between the two ISS divisions is prevented due to the barrier structure. The ISS barrier structure between Tha and Cn was identified using histological stain (B-F) and 7.0T MR (I). The ISS barrier structure was confirmed as myelin with versican (D) using HE (E) and fast blue (K), respectively, in which no neuron or neuroglial cell was found. Nissl staining also showed absence of neurons, indicating that the structure was myelin fiber fascicles (C). Myelin integrity was confirmed by Black Gold staining (B). In the oblique sagittal section of rat brain, the boundaries of Cn., ic. and Tha. were not clear and except for ic., which was composed of the myelinated fibres, no other structure was found between Cn. and Tha. (G). The structure in ic was also confirmed as myelin sheaths by EM(H). There were no gap or tight junctions constructed by cell membranes. (I) shows an MR axial image where the barrier structure between Tha and Cn is evident. (K) shows fast blue staining of a coronal slice, where the barrier structure, stained deep blue, was identified as a myelin fibre tract between the Cn and Tha. (J) shows corresponding axial view images with multi-dimensional and stereoscopic reconstruction. The colour-coding of the track density imaging (DTI) indicates the main local orientation of all fiber tracts in the same slice (red: left-right, green: anterior-posterior, blue: inferior-superior). The divisions of Cn are bordered by the internal capsule, external capsule, corpus callosum, and the wall of the lateral ventricle.

### Statistics

Statistical analyses were performed with SPSS 19.0 software (SPSS Inc., Chicago, IL, USA). The datum follows the normal distribution and are expressed as the mean ± standard deviation (SD). To compare the half-life (t½), clearance rate constant (k’), and the effective diffusion coefficient (D) between the Cn and T groups, independent-sample t-tests were used. The datum follows the normal distribution and are expressed as the mean ± standard deviation (SD).

## RESULTS

### ISF drainage in the deep brain

The barrier to ISF drainage in the deep brain has been previously demonstrated using tracer-based MRI, indicating that the ECS is not highly connected. To further validate the separated ISF drainage in the deep brain, ISF was traced with an extracellular fluorescent probe and its distribution was imaged with LSCM at different time points after probe infusion. Results were compared to those of tracer-based MRI at the same time point. Considering factors that may influence substance transport in brain ECS, the hydrodynamic influence on ISF separation and the stability of the transportation barrier were tested with tracer-based MRI under the condition of the deletion of the water channel protein AQP-4 in the cellular membrane.

**Figure 3.**
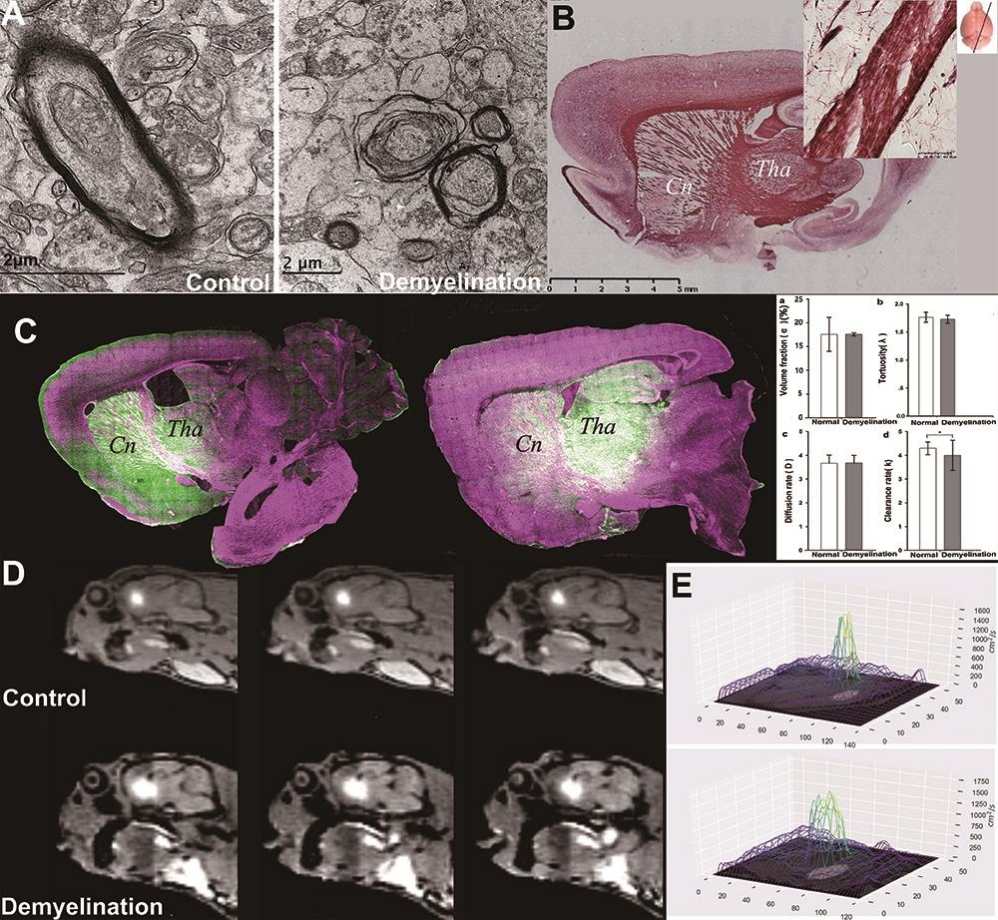
ISF flow is disturbed due to demyelination damage. In the Cuprizon-mediated demyelination rat model, the integrity of the myelin sheath in the internal capsule area was interrupted, as observed using EM and Black Gold staining (B), resulting in myelin sheath splitting, myelin balloon formation and separation from axon. The destruction of the barrier structure accompanied by abnormal ISF flow was observed using LSCM (C). The internal capsule area between Cn and Tha showed demyelination compared to that in the non-demyelination group (A). The traced ISF in one ISS division could be transported to the other (Cn and Tha), i.e., the fluorescent probe in Tha was observed in the adjacent Cn area, and vice versa (C). In the control group, tracer-based MRI showed that the high intensity after Gd-DTPA administration into Cn was limited within the corresponding drainage division and its margin adjacent to the internal capsule was sharp in the control group (upper row, D). No D value could be detected in the D mapping (E). However, in the demyelination group, the high intensity spanned the internal capsule and emerged in Tha (lower row, D). D values could be detected in D mapping. In demyelinated rats (A), communication between the two divisions emerged after the integrity of the myelin sheath in the inner capsule was interrupted, and the ISF in one ISS division could travel to the other. In the Cn division, ISF flow to the cortex was reduced. Thus, local homeostasis was interrupted. Comparison of λ, α, D and k values between control group and demyelination group (columns a-d), clearance rates (k) of the demyelination group were significantly higher (d) (P <0.01), while the others showed no significant difference (a-c) (P>0.05).

Fluorescence imaging was performed with a LY CH probe [16], which was stereotactically injected into the Cn and Tha separately. Brain sampling and scanning were performed with an interval of 0.5 hour. Results were compared to those of the same slice of tracer-based MR [13] images at the same time point (Fig. 1A, B). No significant difference was observed for the maximal distribution ratios between the two groups at different time points (p<0.05) (Fig. 1), which were tested by tracer-based MRI. The transportation barrier to brain ISF flow between Tha and Cn was also demonstrated in normal brains using LSCM. The ISF in the Cn flowed to the ipsilateral frontal cortex. A small amount of fluorescent probe was observed in the adjacent ventriculus tertius after intra-Cn injection, indicating that transport through ependymal cells is also a drainage route for the ISF from the Cn (Fig. 1A). The ISF from Tha drained to both the ventricle and the SAS, but no flow to the Cn was demonstrated. Different structural properties were demonstrated in two different areas (Fig. 1D): a higher volume fraction (α) of ECS was observed in Cn (1.766±0.026) than in Tha (1.673±0.040) (p < 0.01). The tortuosity (λ) of the ECS in Cn (1.173±0.038) was lower than that in Tha (1.352±0.092) (p < 0.05). In addition, a higher diffusion rate with a lower clearance rate was observed in Cn (p < 0.05).

The aquaporin (AQP) family of integral membrane proteins plays a crucial role in regulating water metabolism. AQP-4 is the most abundant bidirectional aquaporin expressed in the brain and is widely distributed on the foot processes of astrocytes. AQP-4 plays a critical role in interstitial flow [10, 17]. In the present study, to test the stability of the transport barrier, AQP-4 knockout rat model was built and it was used to examine the effects of AQP-4 on the transportation barrier [18]. Tracer-based MRI revealed the traced brain ISF flow in the brains of AQP-4 knockout rats (Fig. 1C, Supplementary Fig. 5). In AQP-4-knockout rats, brain ISF could not be transported across the barrier, as in the control group brain. The half-life of the tracer in the brain ISS within the center of the Tha was significantly prolonged in the AQP4 knockout group (0.81±0.03 hours in the Control group and 1.39±0.22 hours in the AQP4^-/-^ group, p<0.05), indicating significantly decreased transportation efficiency (Fig. 1E).

**Figure 4.**
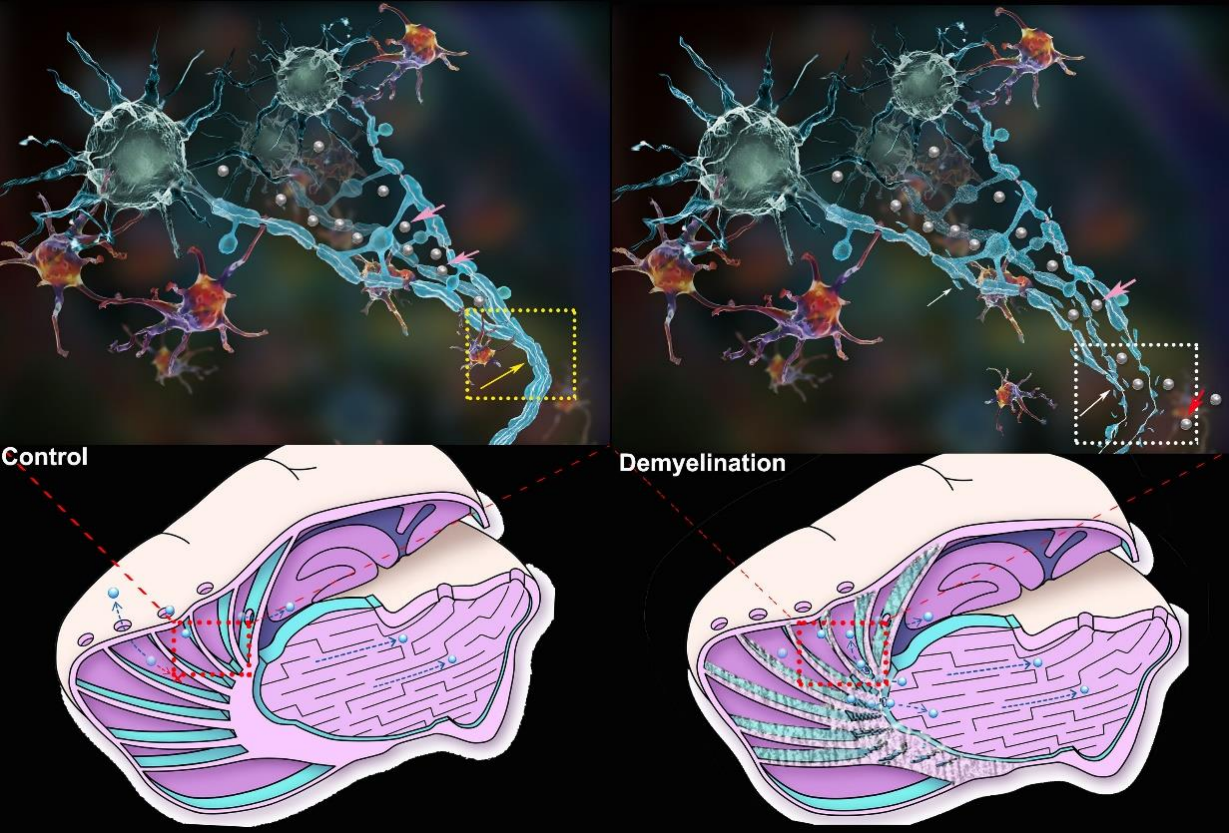
ISF drainage routes in normal rats and demyelinated rats. Due to the obstruction of the compact myelinated fibre tracts (yellow frame and yellow arrow), the traced ISF from the caudate nucleus (Cn)(pink arrow) could not drain to the ECS of the thalamus (Tha) and vice versa, even though these two regions are adjacent to one another. Myelin is identified as the transportation barrier to ISF drainage in the deep brain. At the meantime, it guides the ISF in the caudate nucleus draining to ipsilateral cortex and finally into the subarachnoid space, which maintains the pathway of ISF-CSF exchange. When the integrity of myelin is interrupted (white frame and white arrow), abnormal communication of ISF from the two regions emerges which indicates that the ISF from caudate nucleus (pink arrow) could be drained into the adjacent thalamus (red arrow) and less ISF from caudate nucleus could be drained to the cortex regions. Then the local homeostasis was interrupted. This is a schematic figure for identification purpose and is not be scale.

### Identification of barrier structure to ISF drainage

To identify the structure that blocked the ISF flow between the Cn and Tha, rat brains were scanned with 7.0-T MR *in vivo*. Oblique sagittal sections of the brain (Supplementary Fig. 4) were prepared for EM, Black Gold staining (Fig. 2B), Nissl staining (Fig. 2C), versican staining (Fig. 2D), hematoxylin and eosin (H&E) staining (Fig. 2E), and Luxol fast blue (LFB) staining (Fig. 2F). Histologically, the structure between the Tha and Cn was confirmed to be myelin fibre tracts based on positive LFB staining (Fig. 2F) and the high expression of versican (Fig. 2D). Versican is a large chondroitin sulphate proteoglycan incorporated in the extracellular matrix which surrounds myelinated fibres and accumulates at the nodes of Ranvier. The myelin structure was also confirmed by EM (Fig. 2H). In addition to myelin, the areas bordering the Cn and internal capsule(ic), as well as the ic and Tha, showed no gap or tight junctions constructed by cell membranes. To obtain a gross view of the slice and to determine the distribution of myelin fibres between the Cn and Tha, we scanned the entire slice with high resolution T2-weighted 7T MRI. We observed that the patch body in the striatum was in fact the section view of the corona radiata in the Cn region, which converged to form the internal capsule (Fig. 2G), where substance transport between the Cn and Tha was blocked.

We used MR diffusion tensor imaging to observe the trajectory of the barrier structure which we identified as the internal capsule passing between the Tha and Cn. This arose from the frontal cortex and travelled for long distances across different brain regions (Fig. 2I, J). Axial directionally encoded colour fractional anisotropy maps and gross scans of fast blue stain (Fig. 2K) across the third ventricle slice (bregma: -4.60 mm) showed that the Cn on the slice was well surrounded by the internal capsule, external capsule, corpus callosum, and the lateral ventricle wall.

### Abnormal ISF drainage after myelin damage

To further validate if myelin fibre tracts acted as a barrier to ISF flow, we employed a demyelinated rat model, whereby rats were fed dicyclohexanoneoxalydihydrazone (Cuprizon) for 4 weeks [19]. The demyelied structure was confirmed by EM (Fig. 3A). The flow of the traced ISF was imaged and measured by tracer-based MRI *in vivo* and LSCM (Fig. 3D)*.* Three hours after injection of the fluorescent probe into the Cn or Tha, the rats were sacrificed and fixed by 4% paraformaldehyde. Oblique sagittal brain sections were scanned and imaged by LSCM. Compared to control group (Fig. 1A), the originally compact high reflective fiber tracts showed low reflectivity under LSCM. The demyelination was further verified by Black Gold staining (Fig. 3B). The traced ISF moved across the barrier structure and reached adjacent divisions, as observed on both MRI and LSCM (Fig. 3C). The diffusion rate of the internal capsule was calculated as the tracer’s abnormal diffusion and distribution in the demyelinated rat brain (1.018 × 10^-6^ mm/s; Fig. 3E, Supplementary Fig. 3). The results revealed that the separation effect of the internal capsule was impaired following demyelination of the fibre tracts.

## DISCUSSION

In the present study, ISF drainage routes in the deep brain were revealed using tracer-based MRI and fluorescence imaging. Together with findings on the drainage route of ISF-cerebrospinal fluid (CSF) and the CSF-lymph system, a more detailed picture of ISF drainage pathways in the whole brain can be built [2, 4, 5, 10, 20, 21]. We also demonstrated a novel function of myelin in regulating ISF drainage and substance transport in the brain ECS. Myelin is crucial for supporting axonal metabolism and speeding up electrical conduction along neural pathways [22-24]. In the present study, we observed that the traced ISF moved along the myelin fiber tracts from the deep nuclei to the superficial cortex; and in the opposite direction, the compact myelin fiber fascicles acted as a barrier impeding movement to adjacent ECS divisions. We further demonstrated an abnormal leakage of the traced ISF to the adjacent ECS division in a demyelinated rat brain (Fig.4). Indeed, a similar disturbance has been reported in an aggressive C6 glioma model [25]. Thus, we demonstrate that the brain is protected not only by the blood-brain barrier (BBB), which avoids potential exogenous damage through the vascular system, but is also protected by an internal ISF drainage barrier to avoid potentially harmful interference from other ECS divisions in the deep brain. If the integrity of the barrier is perturbed, local brain function of adjacent ECS divisions may be simultaneously disturbed. Numerous lines of evidence show that normal myelination processes are interrupted in psychosis, aging, Alzheimer’s disease, Parkinson’s disease and other neurodegenerative diseases [26-28]. The discovery of a novel function of myelin in maintaining local brain homeostasis may provide new perspectives for understanding the underlying mechanism of demyelination-related brain disorders. And the newly discovered ECS divisions and regionalized homeostasis may support the notion of many neurosurgeons that “Preservation is the best means of reconstruction. Save every structure”[29]. Further studies on the boundary structure produced by the membrane of oligodendrocytes and the pathological changes of the microenvironment of ECS during demyelination damage will be essential to understand the new function of myelin and its role in maintaining brain function.

The discovery of drainage routes at a whole brain scale will also provide promising strategies for reviving failed central nervous system (CNS) drugs and create new opportunities for drug development, which has long faced a dilemma of low efficiency[30, 31]. Drug administration via ECS is a promising technique that allows therapeutic delivery bypassing the BBB in a targeted and safe manner, and achieves therapeutic drug concentrations more efficiently [14]. A phase III clinical trial of anti-tumor agent delivery via ECS was approved by the Food and Drug Administration of the United States in 2009 [32]. Moreover, prophylactic administration of cytidine diphosphate choline (CDPC) via ECS has recently been proven to be more efficient in protecting neurons against cerebral ischemic injury than routine administration [12]. Notably, we confirmed that transport of small hydrophobic polar molecules in ECS could be modulated by neuronal excitation, which was induced with an external stimulation on the extremities non-invasively [11]. The understanding of the rules of ISF drainage in the deep brain and drainage regulation mechanism will help us to better select the injection location, formulate the administration strategy and improve treatment effect in brain disease treatment. In addition, a new artificial intelligence model based on our findings of the ISF drainage system around neural circuits is warranted, which will explores artificial intelligence field from a new perspective [33].

## Supplementary Materials

The Supplemenantry data can be found online at: www.aginganddisease.org/EN/10.14336/AD.2018.1026
