# Supplementary material for: The Drainage of Interstitial Fluid in the Deep Brain is Controlled by the Integrity of Myelination
Source: Aging Dis. 2019 Oct 1;10(5):937–48. doi: 10.14336/AD.2018.1206 (PMC6764732; doi:10.14336/AD.2018.1206)
Supplement: Supplementary file 1 — The Supplemenantry data can be found online at: www.aginganddisease.org/EN/10.14336/AD.2018.1026 [file AD-10-5-937-s.pdf]

SUPPLEMENTARY DATA

The Drainage of Interstitial Fluid in the Deep Brain is Controlled by the Integrity of Myelination

Aibo Wang[1,2], Rui Wang[1,2], Dehua Cui[2], Xinrui Huang[3], Lan Yuan[4], Huipo Liu[5], Yu Fu[7], Lei Liang[6], Wei Wang[1,2], Qingyuan He[1,2], Chunyan Shi[1,2], Xiangping Guan[1,2], Ze Teng[1,2], Guomei Zhao[1,2], Yuanyuan Li[1,2], Yajuan Gao[2], Hongbin Han[1,2,*]

[1]Department of Radiology, Peking University Third Hospital, Beijing, China. [2]Key Laboratory of Magnetic Resonance Imaging Equipment and Technique, Beijing, China. [3]Department of Biophysics, School of Basic Medical Sciences, Peking University, Beijing, China. [4]Peking University Medical and Health Analysis Center, Peking University Health Science Center, Beijing, China. [5]Institute of Applied Physics and Computational Mathematics, Beijing, China. [6]Department of Medical Chemistry, School of Pharmaceutical Sciences, Peking University, Beijing, China.[7]Department of Neurology, Peking University Third Hospital, Beijing, China.

© 2018. Wang A et al. Published online at http://www.aginganddisease.org/EN/10.14336/AD.2018.1206

SUPPLEMENTARY DATA

1.Tracer-based MRI (NanoDetect Analyze system, MRI lab; Beijing, China (version 2.1))

Stereotaxic intracranial injections of tracer in tracer-based MRI

The paramagnetic tracer, Gd-DTPA (Magnevist; Bayer Schering Pharma AG, Berlin, Germany) or an optic-magnetic bimodal molecular tracer, Gd-DO3A-ethylthiouret-fluorescein (Gd-DO3A-EA-FITC), which was synthesized by our lab, was diluted to 10 mmol/L with 154 mmol/L NaCl solution. After the injection of 2 μL (10 mmol/L) to different regions of the brain, tracer concentration decreased rapidly in a short period of time. These tracers also cannot be uptaken by neuroglial cells. MRI scanning was used to conduct and design the injection route and depth before injection. Each rat was anaesthetized; core temperature was monitored with a rectal thermometer and maintained with a heating pad at 38±0.5°C. Additionally, other physiological variables, such as blood pressure, heart rate and respiratory rate, were monitored; these showed no significant differences between the groups (data not shown). The skin covering the calvaria was shaved and disinfected with iodized alcohol. An incision was made in the scalp along the sagittal suture from the interaural area to the interocular area. The membranes and muscle attachments were dissected free of the skull bone, and the bregma suture was exposed. The rat was immobilized in a stereotactic coordinate system (Lab Standard Stereotaxic-Single, Stoelting Co., Wood Dale, IL, USA) and a small trephine hole was made in accordance with the stereotactic coordinates of Tha (bregma: −3.0 mm, lateral: 2.0 mm, vertical: 6.0 mm) or *Cn* (bregma: +1.0 mm, lateral: 3.5 mm, vertical: 5.0 mm). A 2-μl total volume of the tracer (10 mmol/L) was delivered into the brain interstitial space (ISS) via a 10 μL microsyringe (Hamilton, Bonaduz AG, Switzerland) at a rate of 0.2 μL/min with an automated drug administration system (Harvard Apparatus, Holliston, MA, USA); the infusion was followed by a 5-min waiting period to avoid dorsal reflux along the needle track (Supplement Fig. 1). The rat was then quickly placed in the scanner in a prone position for the post-injection scan, in accordance with the MRI scan protocols.

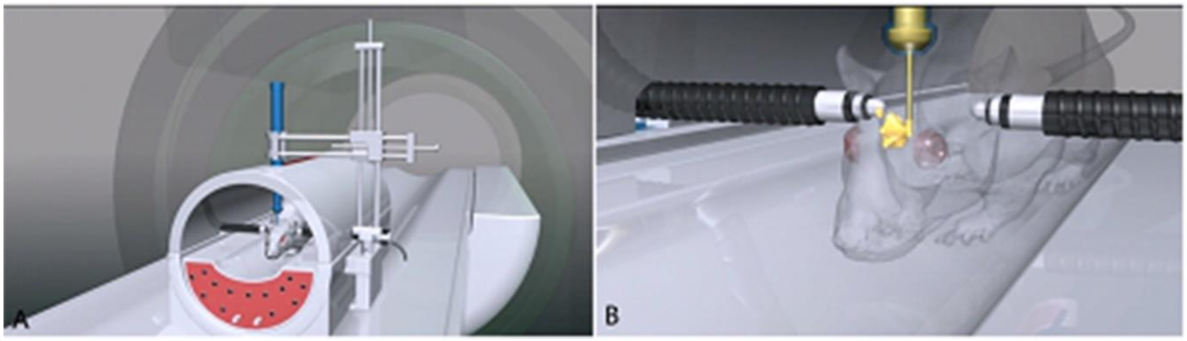

Supplementary Figure 1. Schematic diagram of stereotaxic intracranial injections of tracer in tracer-based MRI. MRI scanning was used to conduct and design the injection route and depth before injection. After the injection of tracer to different regions of the brain, tracer will distribute in the corresponding region and its concentration will decrease rapidly in a short period of time.

MRI scan protocol

A 3.0 T MRI system (Magnetom Trio, Siemens Medical Solutions, Erlangen, Germany) with an eight-channel wrist coil was used to obtain rat brain images by running a T1-weighted magnetization-prepared rapid-acquisition with gradient echo (MP-RAGE) sequence. The acquisition parameters were as follows: echo time = 3.7 ms, repetition time = 1500 ms, flip angle = 12°, inversion time = 900 ms, field of view = 267 mm, voxel = 0.5 mm^3, matrix = 512 × 512, number of averages = 2, phase-encoding steps = 96. The acquisition time for each rat was 290 s. For each subject, scanning was performed before and after the introduction of Gd-DTPA. The scan time points were set as pre-injection and each post-injection time point until the "bright region" faded (Supplement Fig. 2).

Post-procedure calculations of physiological parameters

MATLAB-based software was developed to co-register the MR images of individual rats before and after the injection. All images following the injection were automatically subjected to rigid transformation, similarity measurements, high-order interpolation, and an adaptive stochastic gradient descent optimization. These images were then subtracted from

© 2018. Wang A et al. Published online at http://www.aginganddisease.org/EN/10.14336/AD.2018.1206

the pre-scanned image. The acquired “bright areas”, which were obtained by establishing a seed point, and a threshold in the ROI were assumed to be related to the tracer. New sets of post-processing MR images in the horizontal, sagittal, and coronal planes, with slice thicknesses of 1 mm, were generated by the software. After the co-registration and subtraction processes were completed, signal intensity within the target area of the processed MR images was measured and denoted by ΔSI, which was used to calculate the diffusion parameter in the interstitial space of rat brain (Supplement Fig. 2).

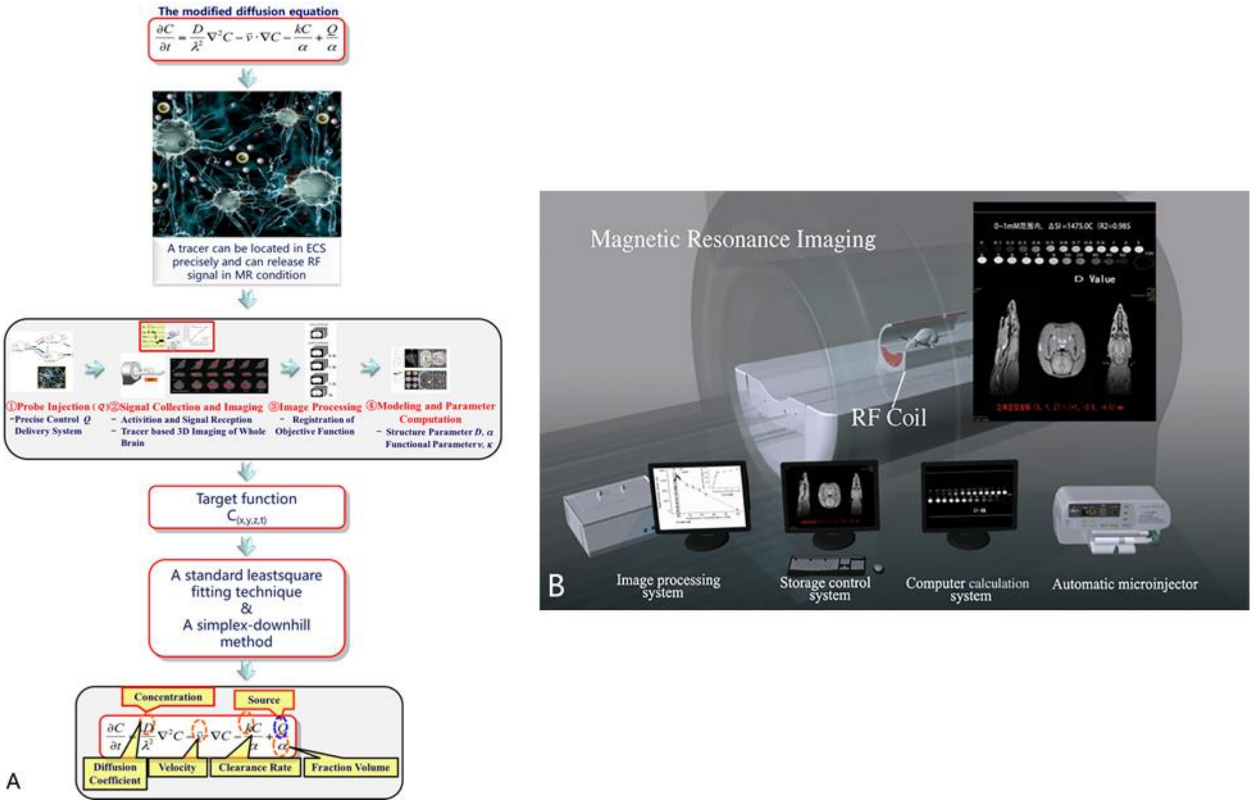

Supplementary Figure 2. The flow chart and working scenario of Tracer-based MRI system. The tracer-based MRI system is consisted of image processing system, computer calculation system. After the tracer injection, brain tissue around the injection site appeared as a high-intensity spot on the MR image. The enhancement of the MR signal intensity caused by the tracer was converted to its concentration by using an empirical fitting process. And by using the modified diffusion equation, the equivalent diffusion coefficient, D, and clearance parameters, k’, the volume fraction α and the tortuosity λ in each MRI pixel near the injection site could be derived. In the present study, the “matching” processes were performed by using the standard least-squares fitting technique and a simplex-downhill method to perform the numerical minimization.

Brain tissue around the injection site appeared as a high-intensity spot on the MR image, shortly after the injection of Gd-DTPA or Gd-DO3A-EA-FITC. The enhancement of the MR signal intensity caused by the tracer was converted to its concentration by using an empirical fitting process. Thus, both the flow and diffusion parameters of the brain interstitial fluid (ISF) could be calculated on the basis of the obtained distribution of the tracer concentration. By using the modified diffusion equation, the equivalent diffusion coefficient, D, and clearance parameters, k’, in each MRI pixel near the injection site could be derived. Here, Vdmax is defined as the ratio of the maximum volume distribution of traced brain ISF to the total rat brain volume, measured by the above-described method. Because the clearance of the tracer in whole rat brain fits well with a mono-exponential decay function, the clearance coefficient (k) and half-life (t½) could be used to represent both clearance rate and transportation speed. This is because substance transportation in the ISS is attributed to diffusion and bulk flow; thus, the clearance rate can be used to depict the flow velocity of the tracered ISF when diffusion velocity is determined.

© 2018. Wang A et al. Published online at http://www.aginganddisease.org/EN/10.14336/AD.2018.1206

SUPPLEMENTARY DATA

After obtaining MR images of the rat brain, we drew a three-dimensional (3D) wireframe by using the "matplotlib" Python package. The height of the figure represents the signal intensity of the MR image. The XY plane at the bottom of the figure is a contour map, superimposed on the original MR image (Supplement Fig. 3). We also produced a 3D transparent model of the rat brain via volume-rendering technology. First, the complete rat brain was artificially segmented from MR images. Then, based on the segmented rat brain MR image, the 3D transparent model was generated using the VTK toolkit. We used red colour to represent the injection area in the model.

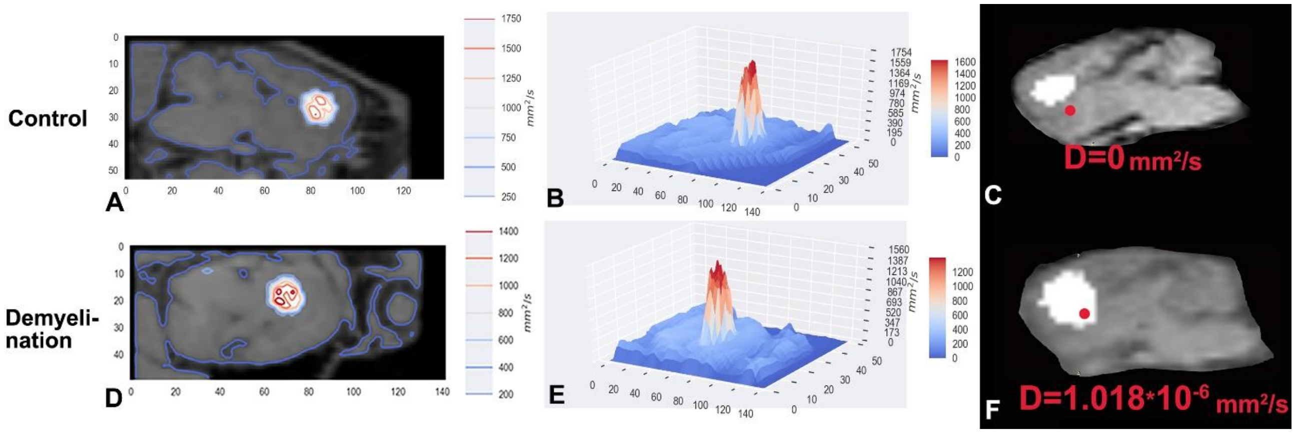

Supplementary Figure 3. Dmapping of tracer-based MRI. After obtaining MR images of the rat brain, we drew a three-dimensional (3D) wireframe, the Dmapping images, by using the "matplotlib" Python package. The height of the figure represents the signal intensity of the MR image. The XY plane at the bottom of the figure is a contour map, superimposed on the original MR image. Dmapping shows the distribution of D values in every voxel and different colors represent different D values. In the pseudo-color images and contour map images, the distributions of tracer in Cn between control group and demyelination group are different. In control group the tracer was limited within the corresponding drainage division and its margin adjacent to the internal capsule was sharp in the control group (A, B). No D value could be detected in the Dmapping (C). However, in the demyelination group, the tracer spanned the internal capsule and emerged in Tha (D, E). D values could be detected in D mapping(F).

Mathematical modelling

Based on the modified diffusion (1) and a standard least-squares fitting technique (2), a program (unpublished) was used for the computation of the diffusion parameters as follows:

$$\frac{\partial C_{\mathrm{ECS}}}{\partial t} = D^{*}\nabla^{2} C_{\mathrm{ECS}} + \frac{Q}{\alpha} - v \cdot \nabla C_{\mathrm{ECS}} - \frac{f\left(C_{\mathrm{ECS}}\right)}{\alpha} \quad (1)$$

where C_{ECS} is the actual concentration in the extracellular space (ECS) and is a function of time (t) and position. The signs ∇ and ∇^2 symbolize, respectively, the first and second spatial derivatives in the appropriate coordinate system. The volume fraction α is defined as the volume fraction of ECS in the whole brain tissue. Tortuosity is defined as $\lambda = (D/D*)^{1/2}$, where D∗ is the effective diffusion coefficient of a given molecule in the brain ECS and D is the diffusion coefficient of the same molecule in a free medium. λ is the hindrance to diffusion, imposed by the local structure of the ECS; Q is the source that is released into the ECS. In the following text, the term "source" refers to the initial concentration available for diffusion or, in the case of an exogenous tracer (e.g., Gd-DTPA), it refers to the site of the injection. Because there was no constant input to the system, Gd-DTPA was assumed to have been administered at the original time point; thus, the source Q can be ignored in the present study. v represents the flow rate of the interstitial fluid in the ECS in mm/s. We set the ROI as close to the injection point as possible (2 mm from the original point), and bulk flow is neglected because of its minimal influence on diffusion. ISF in the local ECS is also assumed to be nondirectional. We further deduced the following equation to facilitate the computation of the diffusion parameters:

$$\chi = \sum_{i=1}^{N} \int_{0}^{r_0} \left(\Delta \mathrm{SI}\left(t_i, r; D^{*}, \beta\right) - \Delta \mathrm{SI}_{,\mathrm{m}}\left(t_i, r\right)\right)^{2} d \quad (2)$$

© 2018. Wang A et al. Published online at http://www.aginganddisease.org/EN/10.14336/AD.2018.1206

Where $\Delta SI_{,m}$ (t_i,r) represents the measured radial profile at time t_i: as a series of MR images are captured at predetermined time intervals, t_i $(i = 1, 2, ..., n)$, the radial profile along a given radial path for each MR image can be measured. The diffusion parameters D^* and β can be measured by matching the model and measured profiles at time points t_i. In the present study, the "matching" processes were performed by using the standard least-squares fitting technique; the function variable χ was minimized to find D^* and β, and the error of the measured profile was assumed to be the same for all data points. In the computation of χ, the integration over radius r was replaced by summation along a given path, which was a particular axis along which the measurements were made from the injection site. A simplex-downhill method was used to perform the numerical minimization.

2. Preparation of oblique brain slices

The oblique sagittal brain slices were prepared to facilitate simultaneous viewing of the caudate nucleus, internal capsule, and thalamus in the same slice. The oblique sagittal slice was rotated 15° outward from the sagittal position (Supplement Fig. 4).

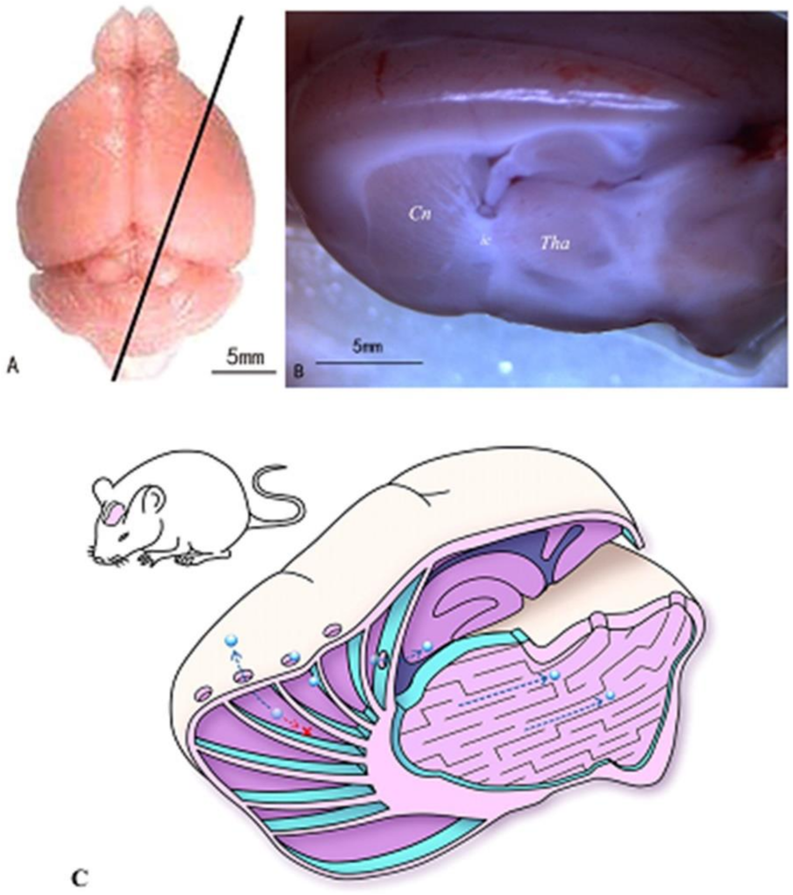

Supplementary Figure 4. The physical photograph and schematic diagram of oblique sagittal brain slices. The oblique sagittal slice was rotated 15° outward from the sagittal position, using the midpoint of the cerebellum as its axis (A). In this section, we could simultaneously observe the caudate nucleus, internal capsule, thalamus, and their associated fibre tracts (B, C). It was also convenient for observing ISF drainage by using tracer-based imaging methods.

© 2018. Wang A et al. Published online at http://www.aginganddisease.org/EN/10.14336/AD.2018.1206

SUPPLEMENTARY DATA

3. Diffusion in AQP4 knockout rats

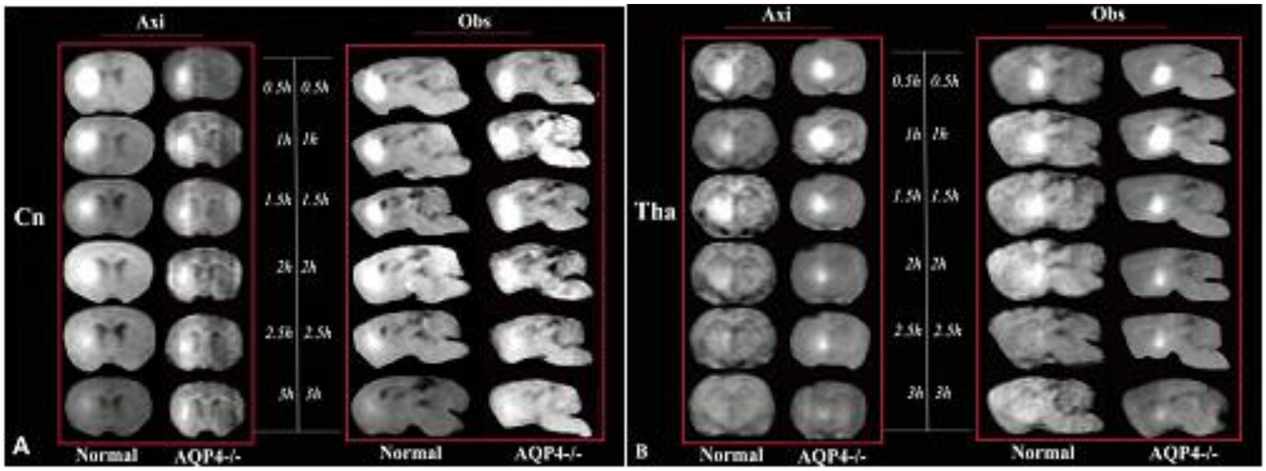

Supplementary Figure 5. Tracer based MRI of normal and AQP4 knockout rats. The continuous dynamic scan of tracer-based MRI in normal and AQP4 knockout rats showed that in AQP-4-knockout rats, brain ISF could not be transported across the barrier, as that in the control group brain. And the clearance rate was obviously decreased in AQP4 knockout rats.

© 2018. Wang A et al. Published online at http://www.aginganddisease.org/EN/10.14336/AD.2018.1206
